# VEGF Treatment Ameliorates Depression-Like Behavior in Adult Offspring after Maternal Immune Activation

**DOI:** 10.3390/cells9041048

**Published:** 2020-04-22

**Authors:** Spyridon Sideromenos, Claudia Lindtner, Alice Zambon, Orsolya Horvath, Angelika Berger, Daniela D. Pollak

**Affiliations:** 1 Department of Neurophysiology and Neuropharmacology, Medical University of Vienna, 1090 Vienna, Austria; spyridon.sideromenos@meduniwien.ac.at (S.S.); alice.zambon@meduniwien.ac.at (A.Z.); orsolya.horvath@meduniwien.ac.at (O.H.); 2 Department of Paediatrics, Division of Neonatology, Pediatric Intensive Care Medicine and Neuropaediatrics, Medical University of Vienna, 1090 Vienna, Austria; claudia.lindtner@meduniwien.ac.at (C.L.); angelika.berger@meduniwien.ac.at (A.B.)

**Keywords:** maternal immune activation, depression, VEGF, tail suspension test, sucrose preference test

## Abstract

Maternal immune activation (MIA) during pregnancy impacts offspring neurodevelopmental trajectories and induces lifelong consequences, including emotional and cognitive alterations. Using the polyinosinic:polycytidilic acid (PIC) MIA model we have previously demonstrated enhanced depression-like behavior in adult MIA offspring, which was associated with reduced expression of the vascular endothelial growth factor (VEGF) receptor 2 (VEGFR2) in the hippocampus. Since VEGF mediates the effects of various antidepressant agents, we here set out to explore whether VEGF administration could rescue the depression-like behavioral deficits in MIA offspring. To test our hypothesis, control and MIA offspring were intracerebroventricularly (i.c.v.) infused with either VEGF or vehicle solution and depression-related behavior was assessed in the sucrose preference test (SPT) and the tail suspension test (TST). As a surrogate of VEGF activity, the phosphorylation of the extracellular signal-regulated kinase (ERK) in hippocampus was quantified. We found that VEGF treatment reduced depression-related behavioral despair in the TST in MIA offspring but had no effect on anhedonia-like behavior in the SPT. While VEGF administration induced the phosphorylation of ERK in the hippocampus of control offspring, this effect was blunted in the MIA offspring. We conclude that VEGF administration, at the dosage tested, beneficially affects some aspects of the depression-like phenotype in the adult MIA offspring, inviting further studies using different dosage regimes to further explore the therapeutic potential of VEGF treatment in MIA-related changes in brain function and behavior.

## 1. Introduction

Depression is a highly debilitating disorder which affects more than 260 million people worldwide [1]. While several risk factors can account for the onset and progression of mood disorders, such as depression, it is the complex interaction between genetic and environmental factors that most likely leads to the clinical manifestation of these disorders [2]. Among the environmental factors, maternal infection during pregnancy constitutes a notable example. The fetal period represents an extremely critical stage for the developing brain and different stressors [3], such as activation of the maternal immune system, can impede normal brain development [4,5]. Consequently, it has been postulated that maternal infections predispose offspring to certain neuropsychiatric disorders, including depression, schizophrenia and autism spectrum disorders [6]. This association was first established by epidemiological studies in the human population [7,8] and recapitulated and extended in various animal models. 

We have previously reported that challenging the immune system of pregnant mice with polyinosinic:polycytidilic acid (PIC), which mimics viral infections, is associated with augmented depression-like behavior in the adult offspring [9,10,11]. 

Neurotrophic factors play a pivotal role in normal brain development and function, and it is not surprising that depression has been closely linked with altered levels of certain neurotrophic factors and is often described as a neurotrophic factor-deficient state [12]. Similar findings have been reported in the brain of maternal immune activation (MIA) offspring [13]. Vascular endothelial growth factor (VEGF) is a neurotrophic factor that binds to VEGF receptor 1 (VEGFR1) and VEGFR2 in the mammalian brain [14]. VEGF is implicated in the regulation of neurogenesis [15] and can mediate, at least in part, the antidepressant effects exerted by both typical antidepressants [16] and ketamine [17,18]. Previous work has shown that MIA-induced enhancement of depression-like behavior and impaired hippocampal dentate gyrus neurogenesis are accompanied by reduced expression of VEGFR2 in the hippocampus of adult offspring [9], suggesting a possible involvement of aberrant VEGF signaling in the observed behavioral deficits. Considering existing evidence for a link between VEGF, neurogenesis and depression, the aim of the current study was to explore whether VEGF administration could rescue the depression-like behavioral phenotype of adult MIA offspring. 

We report that VEGF ameliorated the behavioral deficits present in the tail suspension test (TST) but had no effect in the sucrose preference test (SPT). At the dose tested, VEGF did not exert any behavioral effects in control offspring. Furthermore, while VEGF increased phosphorylation of extracellular signal-regulated kinase (ERK) in the hippocampus of control offspring, this effect was blunted in the MIA offspring, further extending insights into the impairment of VEGF signaling in the MIA progeny and inviting future studies for the exploration of the therapeutic potential of VEGF in the MIA model of depression.

## 2. Materials and Methods

### 2.1. Animals 

All mice used were C57Bl6/N obtained from Charles River (Sulzfeld, Germany). All animal experiments were conducted in agreement with the ARRIVE guidelines and the U.K. Animals (Scientific Procedures Act, 1986 and associated guidelines, EU Directive 2010/63/EU for animal experiments) and approved by the national ethical committee on animal care and use (Bundesministerium für Wissenschaft und Forschung: BMBWF-66.009/0175-V/3b/2019). Mice were kept at the local animal facility under standard laboratory conditions with 12:12 h light:dark cycle with food and water available ad libitum. 

### 2.2. Breeding

Breeding of MIA and control offspring has been previously described [9]. Briefly, after overnight mating the presence of vaginal plugs was recorded the following morning and this day was considered embryonic day (E) 0.5. At E12.5 pregnant mice were randomly selected for intraperitoneal injection (i.p.) with either 0.9% NaCl or 20 mg/kg polyinosinic:polycytidylic acid (PIC) (Lot: 096M4023V, Sigma Aldrich, St. Louis, USA) potassium salt. The injection volume was 10 mL/kg bodyweight in each case. The administration protocol was chosen based upon the authors’ own previous studies demonstrating behavioral and neural alterations relevant for depression in the adult MIA offspring [4,9,11,19]. A detailed maternal immune activation checklist [20] is provided in Supplementary Table S1. 

Offspring were weaned at 3 weeks of age, separated by sex and kept in cages with same-sex littermates until adulthood. Adult male offspring (12 weeks of age at the onset of experiments) were included in the present study. All animals were single-housed two weeks before the onset of experiments.

### 2.3. Pump Implantation

Mini-pumps (1007D, AZLET) and brain infusion equipment (Brain infusion kit 3, AZLET, VWR, USA) were connected following the manufacturer’s instructions. Pumps were filled with rhVEGF (293-VE-010, R&D, Minnesota, USA) reconstituted in artificial cerebrospinal solution (aCSF) (preparation of aCSF according to manufacturer’s instructions, Azlet, VWR, USA) or aCSF alone. Mice were deeply anesthetized with isoflurane and the cannulae were inserted into the left lateral ventricle (AP: –0.3 mm; ML: 1.25 mm; DV: 3 mm). The osmotic mini-pumps were carefully implanted subcutaneously in the back of the animals. Immediately after surgery, mice received an i.p. injection of tramadol hydrochloride (25 mg/kg, Tramal, Grünenthal, Aachen, Germany). Carprofen (0.15 mg/mL, Rimadyl, Zoetis, New Jersey, USA) was dissolved in the drinking water and administered for 2 more days. Pumps contained a solution of 2.5 μg/mL of rhVEGF or aCSF alone. The infusion rate was 0.5 μL/h, resulting in a dose of 30 ng/day. Previous studies have utilized a dosage of 240 ng/day [15] in rats. Accordingly, since the cerebrospinal fluid volume in mice [21] is approximately 7–8 times smaller than in rats [22], the dose employed here corresponds to 12% of the dose previously used in rats.

### 2.4. Behavioral Analysis

**Figure 1:**
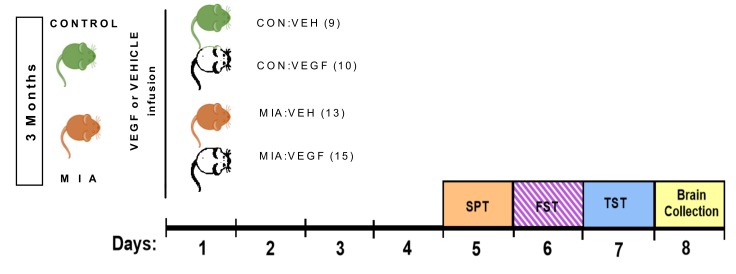
Schematic overview of the experimental design. MIA: maternal immune activation; VEH: vehicle; VEGF: vascular endothelial growth factor; SPT: sucrose preference test; FST: forced swim test; TST: tail-suspension test. Numbers in parentheses indicate the number of animals used in each group.

Behavioral testing began after a recovery period of 4 days (Figure 1). We used three standard behavioral paradigms to evaluate depression-like behavior in MIA and control mice and its response to VEGF treatment: the SPT, the forced swim test (FST) and the TST. However, as explained in detail in the Discussion section, results of the FST were not considered further for data analysis and interpretation. The SPT is typically considered to indicate depression-related anhedonia, while the FST and the TST assess depression-related behavioral despair. [23]. 

#### 2.4.1. Sucrose Preference Test 

The SPT was conducted as previously described [9], with minor modifications. The training session occurred 4 days before the pump implantation. Mice were food- and water-deprived for 18 h, after which they were exposed to food and a single bottle containing 2% sucrose solution for 48 h. The position of the bottle was altered every 24 h to avoid the establishment of a side preference. The preference test occurred 5 days after the surgery. Mice were food- and water-deprived for 18 h before the 3 h testing period, which consisted of the simultaneous presentation of two drinking bottles: one containing normal tap water and the other 2% sucrose solution. The volume of liquid consumption from each bottle was recorded and used for the calculation of sucrose preference = volume of sucrose consumed/(volume of sucrose consumed + volume of water consumed), as an indicator of depression-related anhedonia.

#### 2.4.2. Tail-Suspension Test 

The TST was carried out according to a published procedure [24] using an automated testing system (MedAssociates Inc., St. Albans, VT, USA). The percentage of immobility during the total period (6 min) test was calculated and used as an indicator of depression-related behavioral despair.

#### 2.4.3. Forced Swim Test (FST)

The procedure for the FST followed a published protocol [9]. However, a significant number of mice in both treatment groups presented with aberrant swimming behavior, and the analysis of immobility in the FST was therefore not considered a reliable parameter for the present study design. 

### 2.5. Western Blots 

Relative phospho-ERK (pERK) and total ERK (tERK) levels were determined by Western blotting according to standard procedures. Briefly, mice were sacrificed by cervical dislocation and brains were quickly extracted, embedded in Optimal Cutting Temperature (O.C.T.; Tissue-Tek, Sakura, Staufen, Germany), rapidly frozen in liquid nitrogen and stored at −80 °C until further processing. Hippocampal tissue was dissected and processed as previously described [11]. Protein samples (25 μg) were subjected to SDS-PAGE electrophoresis with subsequent blotting onto Polyvinylidene fluoride (PVDF) membrane (MilliporeSigma, Massachusetts, USA). The primary antibodies used were: phospho-ERK (4376, 1:1000, Cell Signalling, Denver, USA), total ERK (14-9108-82, 1:1000, Invitrogen, California, USA) and Glyceraldehyde-3-phosphate dehydrogenase (GAPDH) (MA5-15738, 1:3000, Invitrogen, California, USA). Primary incubation was performed at 4 °C overnight and then the membrane was incubated with an appropriate secondary antibody for 2 h at room temperature. Quantification was conducted using the FluorChem HD2 system (Alpha Innotec, Kasendorf, Germany). The membrane was stripped after each quantification using a stripping buffer (Restore™ PLUS Western Blot Stripping Buffer, Invitrogen). Quantification of optical densities was performed with FluorChem HD2 software (Alpha Innotec, Kasendorf, Germany). The levels of phospho-ERK and ERK were expressed relative to GAPDH levels.

### 2.6. Statistical Analysis

All statistical analyses were carried out using GraphPad Prism 7 (La Jolla, California, USA). A two-way analysis of variance (2WAY-ANOVA) with MIA and VEGF as main factors was used to compare the means of the different groups. If significant main effects were found, the Fischer’s least significant difference (LSD) post-hoc test was employed. The Tukey’s box plot method for the identification of statistical outliers was applied. A single outlier was detected and removed (SPT: control-vehicle group (CON:VEH). *P* values < 0.05 were considered significant in all instances. 

A summary of all statistical results is provided in Supplementary Table S2. 

## 3. Results

Control and MIA male offspring were implanted with an osmotic mini-pump, delivering either VEGF or vehicle solution directly to the lateral ventricles of the brain at a dose of 30 ng/day for a period of one week. This resulted in a 2 × 2 design with four experimental groups: Control offspring receiving vehicle (CON:VEH) or VEGF solution (CON:VEGF) and MIA offspring receiving vehicle (MIA:VEH) or VEGF solution (MIA:VEGF). A brief experimental overview is given in Figure 1.

### 3.1. Behavioral Effects of VEGF Infusion

**Figure 2:**
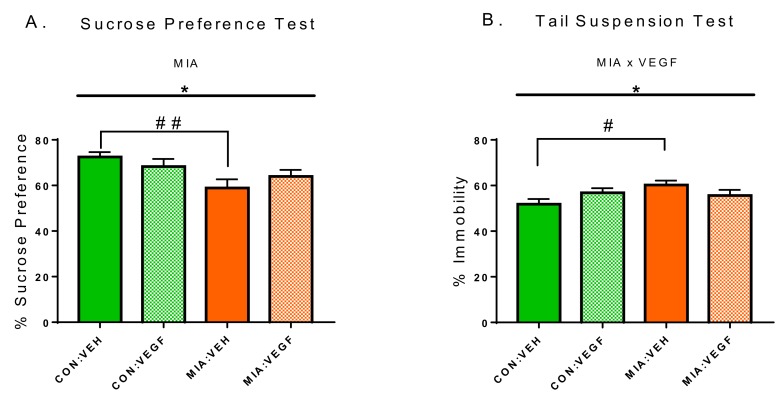
Behavioral effects of VEGF infusion in control (CON) and MIA offspring. (**A**) Percentage of sucrose preference in the sucrose preference test (SPT). (**B**) Percentage of immobility in the tail-suspension test (TST). Data presented as mean ± SEM. Statistical significances resulting from 2WAY-ANOVA are marked with * (*p* < 0.05). Statistical significances resulting from Fisher’s least significant difference (LSD) test are marked with # (*p* < 0.05); ## (*p* < 0.01); *n* = 8–15/group.

We employed the SPT to measure anhedonia, a core symptom of depression in people. Two-way analysis of variance (2WAY-ANOVA) revealed a main MIA effect (MIA, F (1, 42) = 7.043, p = 0.0112, 2WAY-ANOVA, Figure 2A), indicating that VEGF treatment did not ameliorate the known decrease in sucrose preference in MIA offspring [9]. Post-hoc analysis showed significant differences between the CON:VEH and MIA:VEH groups (Fischer’s LSD, *p* = 0.0093, Figure 2A) but not between the CON:VEH and MIA:VEGF group (Fischer’s LSD, *p* = 0.0866, Figure 2A).

To assess depression-related behavioral despair, we initially sought to employ the FST as previously differences have been reported in MIA offspring in this paradigm [9]. However, several mice in both experimental groups displayed aberrant swimming behavior, likely related to the mini-pump implantation. Therefore, we decided to use the TST as an alternative test assessing behavioral despair, as reflected in the percentage of immobility. A significant main interaction effect (MIAxVEGF, F (1, 43) = 4214, *p* = 0.0462, 2WAY-ANOVA, Figure 2B) and post-hoc analysis showed differences only between the CON:VEH and MIA:VEH groups (Fischer’s LSD, *p* = 0.0174, Figure 2B), suggesting that VEGF administration selectively modulated despair-like behavior in MIA offspring.

### 3.2. VEGF Signaling in the Offspring Hippocampus

**Figure 3:**
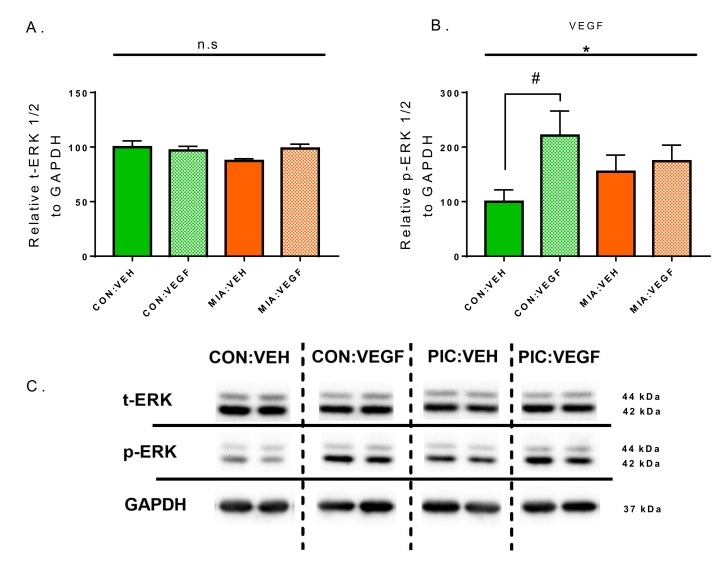
Quantification of total ERK (t-ERK) and phospho-ERK (p-ERK) levels by Western blot. (**A**) Relative total ERK to GAPDH. (**B**) Relative p-ERK to GAPDH. (**C**) Representative Western blot images. Data are normalized to the CON:VEH group for comparison and presented as mean ± SEM. Statistical significances resulting from 2WAY-ANOVA are marked with * (*p* < 0.05). Statistical significances resulting from Fisher’s LSD test are marked with # (*p* < 0.05); *n* = 9–10/group.

Binding of VEGF to VEGFR stimulates the MAPK/ERK signaling cascade [25]. To biochemically monitor the activity of the exogenously administered VEGF, we evaluated ERK 1/2 expression and phosphorylation as proxy in hippocampal tissue of all groups [25,26]. Relative phospho-ERK (pERK) and total ERK (tERK) levels were determined by Western blotting. Quantification of specific bands revealed no group differences for tERK (Figure 3A), while for pERK a significant VEGF main effect was detected (F (1, 34) = 4634, *p* = 0.0385, 2WAY-ANOVA, Figure 3B). However, post hoc pairwise comparisons demonstrated a significant difference between CON:VEH and CON:VEGF (Fischer’s LSD, *p* = 0.0151) but not in MIA:VEH versus MIA:VEGF groups (Figure 3B), indicating that VEGF infusion increased the phosphorylation of ERK solely in control offspring, while in MIA progeny the effect of VEGF on ERK phosphorylation was blunted.

## 4. Discussion

Mood disorders are considered to be the result of a complex interaction between genetic and environmental factors leading to the development of the disorder [2]. Maternal infections during pregnancy represent a well-characterized environmental risk factor for the development of mood disorders, including depression, later in life in people and comparable findings have been obtained in animal models [6,9]. Several brain regions are implicated in the neural circuitry of mood disorders. A prominent role for the hippocampus has been postulated, mainly related to the demonstration of significant atrophy and aberrant neurogenesis in the subgranular zone of the dentate gyrus in depressed patients and animal models [27,28,29,30]. Both findings have been suggested to relate to insufficient neurotrophic support [31]. Among the neurotrophic factors involved in depressive disorders, brain-derived neurotrophic factor (BDNF) has gained much attention [32,33]. Nonetheless, an important role for the regulation of neurogenesis and emotional behavior has also been described for other neurotrophic factors such as VEGF and insulin-growth factor 1 (IGF-1) [34]. With regards to VEGF, it has been demonstrated that VEGF administration promotes neurogenesis [15] and mediates the behavioral effects of both typical antidepressants [16] and ketamine [17].

In this study we explored whether VEGF administration can rescue the behavioral deficits present in MIA offspring. The rationale for these experiments was based on our previous work, showing that MIA offspring display depression-like behavior alongside reduced hippocampal neurogenesis and decreased hippocampal expression of the VEGFR2 [9].

Here, we confirmed the previously reported behavioral effects of MIA on sucrose preference [9,11] and found that VEGF application, in the current paradigm of administration, did not affect performance in the SPT, in either MIA or control offspring. To the best of our knowledge, no direct effect of VEGF administration on sucrose preference has been shown to date, albeit previous studies have reported that blockage of VEGF signaling abolishes the effects of fluoxetine [35] and desipramine [16] in the SPT, suggesting that VEGF is required for the activity of antidepressant drugs on anhedonic behavior but not sufficient to exert an effect by itself. 

In order to complement the observations in the SPT with a second paradigm assessing depression-like behavior we initially employed the FST, where we had previously reported impaired performance in MIA offspring [9]. However, as apparently the mini-pump/brain infusion system interfered with the ability of mice to swim properly in the FST (for reasons not further investigated here), we turned towards the TST, similarly assessing behavioral despair. To eliminate any confounding effects of behavioral testing in the FST itself on subsequent behavioral and/or biochemical evaluations we ensured that each mouse was subjected to the FST, even though the immobility time was not further considered for data analysis. In the TST, VEGF infusion selectively ameliorated the despair-related immobility of the MIA offspring, without affecting the behavior of the controls. Previous studies have shown an effect of VEGF administration on behavior in the FST in rats [16,17] and that constitutive genetic VEGF overexpression in mice resulted in decreased immobility in the FST [36]. However, none of the above-described approaches are directly comparable to the design of the present investigation in consideration of species [16,17], mode of administration/expression [36] and behavioral test employed [16,17] which may explain the distinct phenotypes observed. Hence, a limitation of the current study is that the results obtained have to be considered specific for the mode and protocol of administration and dosage tested herein. Extensions to other experimental conditions to allow for a greater generalization and broader interpretation of the present observations are therefore desirable.

Importantly, earlier reports demonstrated that VEGF administration increases levels of pERK in the brain [15,25], suggesting central pERK as a surrogate of the activity of exogenously administered VEGF. Evaluating tERK and pERK in the hippocampus of VEGF-treated mice we confirm that there are no differences in tERK levels but that VEGF administration induced the phosphorylation of ERK, selectively in the control group. These results confirm previous studies but also indicate that the VEGF-induced phosphorylation of ERK is blunted in the MIA progeny. The present observation implies that the reduction of VEGFR2 in the MIA hippocampus may constitute a limiting constraint for the biochemical effects of exogenously applied VEGF on the stimulation of the ERK signaling pathway. Additionally, one has to consider that VEGFR is expressed in neuronal, glial and endothelial cells. The herein used approach is not suitable for detecting cell-type-specific activation of VEGFR, which may selectively contribute to individual brain/behavioral functions. As such, one could speculate that the selective effect of VEGF on behavioral despair in MIA offspring may be the consequence of stimulation of a specific population of non-neuronal cells, which secrete other factors, such as BDNF [37], that then modulate neuronal activity and consecutively behavioral function. 

## 5. Conclusions

Collectively we here provide first evidence that VEGF administration in adulthood ameliorates some aspects of the depression-related behavioral deficits present in MIA male offspring. This is in accordance with previous studies reporting beneficial effects of VEGF on neuronal mechanisms related to the pathophysiology of depression. These results invite further studies to extend insights into the basic mechanisms and translational potential of the therapeutic relevance of VEGF in the MIA model of gestational infection.

## Supplementary Materials

The following are available online at https://www.mdpi.com/2073-4409/9/4/1048/s1, Table S1: Reporting checklist for maternal immune activation (MIA), Table S2: Full summary of statistical results.
