# Supplementary material for: VEGF Treatment Ameliorates Depression-Like Behavior in Adult Offspring after Maternal Immune Activation
Source: Cells. 2020 Apr 22;9(4):1048. doi: 10.3390/cells9041048 (PMC7226255; doi:10.3390/cells9041048)
Supplement: Supplementary file 1 [file cells-09-01048-s001.pdf]

Supplementary Table S1: Reporting checklist for maternal immune activation (MIA).

**a. Compound**	
Name of compound:	Polyinosinic:polycytidylic acid (Sigma-Aldrich)
Catalogue number:	P9582
Lot number:	096M4023V
Vehicle control used:	0.9% physiological NaCl
Route of administration:	intraperitoneal
Volume administered:	20mg/kg (10µL/g)
Storage conditions:	−20 °C
b. Housing variables at injection	
Temperature of room at injection time:	23,2 °C
Light cycle of animal housing room:	light/dark 12:12
Time of day of injection:	9:00–11:00 AM
Did a cage change occur at time of injection:	no
c. Validation of immune activation	
sickness behavior scoring 2 h and 4 h after injection	
d. Validation of gestational timing	
timed mating and vaginal plug	
e. Animals	
Species:	Mouse
Strain:	C57Bl6/N
Maternal physiological variables at time of immune challenge:	
maternal Age at challenge:	11/12 weeks
maternal Body weight:	~30,1g (average weight)
Vendor:	Charles River Laboratories, Sulzfeld Germany
f. Caging systems	
at breeding: IVC	
Material of cage:	plastic
Cage dimensions:	19.56 × 30.91 × 13.34 cm
after parturition: open cages	
Material of cage:	plastic
Cage dimensions	19.56 × 30.91 × 13.34 cm
after weaning: IVC	
Material of cage:	plastic
Cage dimensions:	19.56 × 30.91 × 13.34 cm
g. Animal Holding Room	
Temperature in room:	22–24 °C
Humidity in room:	~ 50%
Ventilation system:	IVC or open cages
Specific pathogen free [SPF]:	no
Are males & females housed in the same or separate rooms:	same room, but separate areas
h. Bedding exchanges/bedding type	
Type of cage bedding used:	Premium scientific bedding (SAFE)
Frequency of cage changes per week:	
prior to mating and after weaning:	once
during gestation:	twice
during neonatal period:	no cleaning till postnatal day 7
i. Breeding	
timed pregnancy on site	
are the same fathers breeding with both experimental and control dams?	NO
Biological age at shipping:	7/8 weeks
Biological age of dams:	9/10 weeks
Number of Dams bred:	20
How many times have dams been mated previously:	NONE

How many times did the dams mate and not become pregnant:	NONE
Are the dams primiparous or multiparous?	Primiparous
What was the frequency of maternal handling during the gestational/neonatal period:	twice a week
Biological age of sires:	9–18 weeks
Number of sires bred:	10
How many times have sires been mated previously:	0–4
How many times did the sires mate successfully:	once
If bred previously, what was the interval between mating times:	one month
Are sires matched to experimental and control dams:	yes
Describe the mating design (1:1, 1:2 etc):	1:2
j. Social enrichment	
Number of cage companions prior to breeding:	3–5
Gestational age when dam separated for breeding:	9/10 weeks
Number of cage companions at weaning:	2-5
k. Physical enrichment	
Type and quantitiy of enrichment device:	1 paper towel/ cage
l. Details on animal numbers and distribution	
Maternal- N vs offspring N	
total number of -dams/litters included in the study:	
total number of offspring per litter included in the study:	
Litter size and sex distribution	
What size was each litter maintained at:	6–11 (total number)
How many males and females were maintained in each litter:	
How many offspring per litter were used in each measure:	
Randomization/Matching procedures:	randomization
Sex as a biological variable:	only males used
Age(s) of offspring at behavioral testing/ physiological evaluation:	
Unit of analysis for each data set:	based on number of animals

Supplementary Table S2: Full summary of statistical results.

Figure	Experiment	Parameter	Statistical Test	n / group	Factor	Statistics, Degrees of Freedom	*P* value	Multiple Comparisons	P value Fischer's LSD
2A	Sucrose Preference Test (SPT)	Sucrose Preference	2-way ANOVA	8–15				CON:VEH vs. CON:VEGF	0.4248
					VEGF x MIA	F (1, 42) = 1,912	*P* = 0,1740	CON:VEH vs. MIA:VEH	0.0093
					VEGF	F (1, 42) = 0,01519	*P* = 0,9025	CON:VEH vs. MIA:VEGF	0.0866
					MIA	F (1, 42) = 7,043	*P* = 0,0112	CON:VEGF vs. MIA:VEH	0.0515
								CON:VEGF vs. MIA:VEGF	0.35
								MIA:VEH vs. MIA:VEGF	0.2344
2B	Tail-Suspension Test (TST)	% Immobility	2-way ANOVA	9–15				*CON:VEH vs. CON:VEGF*	0.1735
					VEGF x MIA	F (1, 43) = 4,214	*P* = 0,0462	*CON:VEH vs. MIA:VEH*	0.0174
					VEGF	F (1, 43) = 0,006874	*P* = 0,9343	*CON:VEH vs. MIA:VEGF*	0.2552
					MIA	F (1, 43) = 2,402	*P* = 0,1285	*CON:VEGF vs. MIA:VEH*	0.3048

								CON:VEGF vs. MIA:VEGF	0.7155
								MIA:VEH vs. MIA:VEGF	0.129
3A	t-ERK 1/2 (Total ERK levels)	Relative t-ERK 1/2 levels	2-way ANOVA	9–10					
					VEGF x MIA	F (1, 34) = 3,284	*P* = 0,0788		
					VEGF	F (1, 34) = 1,076	*P* = 0,3068		
					MIA	F (1, 34) = 1,882	*P* = 0,1791		

Figure	Experiment	Parameter	Statistical test	n / group	Factor	Statistics, degrees of freedom	*P* value	Multiple comparisons	P value Fischer's LSD
3B	p-ERK 1/2 (Phospho-ERK 1/2 levels)	Relative p-ERK 1/2 levels	2-way ANOVA	9–10				*CON:VEH vs. CON:VEGF*	0.0151
					VEGF x MIA	F (1, 34) = 2,437	*P* = 0,1278	*CON:VEH vs. MIA:VEH*	0.2418
					VEGF	F (1, 34) = 4,634	*P* = 0,0385	*CON:VEH vs. MIA:VEGF*	0.1168
					MIA	F (1, 34) = 0,01527	*P* = 0,9024	*CON:VEGF vs. MIA:VEH*	0.1605
								CON:VEGF vs. MIA:VEGF	0.3166
								MIA:VEH vs. MIA:VEGF	0.6701
								P values < 0.05 were considered significant	
